# The association between albumin and C-reactive protein in older adults

**DOI:** 10.1097/MD.0000000000034726

**Published:** 2023-08-25

**Authors:** Yiqian Jiang, Zhenli Yang, Qinghua Wu, Jianhua Cao, Tiefeng Qiu

**Affiliations:** a Department of Radiotherapy, Xiaoshan Affiliated Hospital of Wenzhou Medical University, Hangzhou, Zhejiang, China; b Department of Gynecology, Xiaoshan Affiliated Hospital of Wenzhou Medical University, Hangzhou, Zhejiang, China; c Department of Radiology, Xiaoshan Affiliated Hospital of Wenzhou Medical University, Hangzhou, Zhejiang, China.

**Keywords:** adults, albumin, CRP, elderly, NHANES, relationship

## Abstract

Albumin had been found to be a marker of inflammation. The purpose of our study was to investigate the relationship between albumin and C-reactive protein (CRP) in 3579 participants aged 60 to 80 years from the National Health and Nutrition Examination Survey (NHANES). In order to evaluate the association between albumin and CRP, We downloaded the analyzed data (2015–2018) from the NHANES in the United States, and the age of study population was limited to 60 to 80 years (n = 4051). After exclusion of subjects with missing albumin (n = 456) and CRP (n = 16) data, 3579 subjects aged 60 to 80 years were reserved for a cross-sectional study. All measures were calculated accounting for NHANES sample weights. We used the weighted χ2 test for categorical variables and the weighted linear regression model for continuous variables to calculate the difference among each group. The subgroup analysis was evaluated through stratified multivariable linear regression models. Fitting smooth curves and generalized additive models were also carried out. We found albumin negatively correlated with CRP after adjusting for other confounders in model 3 (β = −0.37, 95% CI: −0.45, −0.28, *P* < .0001). After converting albumin from a continuous variable to a categorical variable (quartiles), albumin level was also negatively associated with serum CRP in all groups (*P* for trend < .001 for each). In the subgroup analysis stratified by gender, race/ethnicity, smoking, high blood pressure, the negative correlation of albumin with CRP was remained. We also found that the level of CRP further decreased in other race (OR: −0.72, 95% CI: −0.96, −0.47 *P* < .0001) and participants with smoking (OR: −0.61, 95% CI: −0.86, −0.36 *P* < .0001). Our findings revealed that albumin levels was negatively associated with CRP levels among in USA elderly. Besides, CRP level decreased faster with increasing albumin level in other race and participants with smoking. Considering this association, hypoalbuminemia could provide a potential predictive biomarker for inflammation. Therefore, studying the relationship between albumin and CRP can provide a screening tool for inflammation to guide therapeutic intervention and avoid excessive correction of patients with inflammation.

## 1. Introduction

Continued population aging will cause the population over 60 to double in the next few decades.^[1]^ There is increasing evidence that inflammation is a widespread problem in this section of the population.^[2–4]^ As the population ages, the number of older individuals with inflammation will increase dramatically in the coming decades.^[2]^ Because of the medical costs, morbidity, and mortality associated with inflammation, understanding the markers and risk factors for inflammation is essential for the prevention, early diagnosis, and management of inflammation. C-reactive protein (CRP) levels are known to increase dramatically in response to inflammation and it increases in circulation during inflammatory events.^[5,6]^ Therefore, it is well established that CRP is the marker of inflammation that can be measured.

Caloric–proteic malnutrition is also a common disease in the elderly,^[7,8]^ and albumin is considered to be an important indicator of nutritional status.^[7,9,10]^ Meanwhile, albumin is the most widely studied protein for diagnosing malnutrition, and the definition of hypoalbuminemia is used as an indication of malnutrition to screen malnourished people.^[11]^ Albumin plays an important role in a number of physiological mechanisms including the inflammation and nutrition. Furthermore, albumin regulates the osmotic pressure and is a carrier of poorly water-soluble molecules such as calcium, iron, bilirubin, hormones, cholesterol, free fatty acids, and drugs.^[12–14]^ Significant loss of muscle mass has been observed in elderly people with low albumin levels. Hypoalbuminemia is a mortality prognostic factor in elderly people, whether they live in the community or they are in hospital or institutionalized. Low levels of albumin are associated to worse recovery following acute pathologies.^[15]^ There is growing evidence that serum albumin is a negative acute phase protein, which supports the view that plasm albumin is a marker of inflammation.^[16]^

Recently, the focus of the research is the relationship between albumin and CRP. However, a controversial finding was reported in the limited evidences. Specifically, the decrease of albumin was accompanied by a significant increase in CRP.^[16,17]^ Nevertheless, other study reported that albumin had been observed to increase in inflammatory states.^[18]^ Therefore, the purpose of our study was to use a representative sample from the National Health and Nutrition Examination Survey (NHANES) to assess the association between albumin and CRP in elderly. This study included a representative sample of a multi-ethnic population, while a large sample size enabled us to conduct a subgroup analysis, which, to our knowledge, was the first study to evaluate the correlation between albumin and CRP in different multivariate logical regression models.

## 2. Materials and methods

### 2.1. Study population

In our study, the analyzed data were obtained from the NHANES (2015–2018), NHANES is a representative survey of the national population of the United States, which uses a complex, multi-stage, probability sampling design to provide a large amount of information about the nutrition and health of the general United States population.^[19]^ We combined a total of 19,225 sample sizes representing the 2 cycles of NHANES from 2015 to 2018. The age limit of the study population is between 60 and 80 years old, and after excluding patients with severe diseases such as malignant tumors or trauma, 4051 samples meet the research requirements. After again exclusion of subjects with missing albumin (n = 456) and CRP (n = 16) data, 3579 subjects aged 60 to 80 years were reserved for a cross-sectional study.

The National Center for Health Statistics ethics review board approved all NHANES protocols, and participants or their proxies provided informed consent prior to participation.^[20]^ Therefore, these data are publicly available and do not require further ethical review.

### 2.2. Variables

The principal variables of this study were albumin (independent variable) and CRP (dependent variable). The albumin concentration was measured using the DcX800 method which was as a bichromatic digital endpoint method. CRP was measured on the Beckman Coulter UniCel DxC 600 Synchron and the Beckman Coulter UniCel DxC 660i Synchron Access chemistry analyzers. In addition, the following covariates were included: age, alanine aminotransferase, blood urea nitrogen, creatinine, Gamma Glutamyl Transferase, body mass index, alkaline phosphatase, aspartate aminotransferase, cholesterol, creatine phosphokinase, globulin, lactate dehydrogenase, triglycerides, uric acid, total bilirubin, race/ethnicity, martial status, smoking, high blood pressure, gender. Details of albumin and CRP measurement process and other covariate acquisition process are available at www.cdc.gov/nchs/nhanes/.

### 2.3. Statistical analyses

All estimates were calculated accounting for NHANES sample weights. The missing values of categorical variables are grouped separately, and the missing values of continuous variables are replaced by the average values. Study participants are divided into quartiles based on albumin levels. All statistical analyses were conducted using the statistical software EmpowerStats (http://www.empowerstats.com) and packages R (http://www.R-project.org), with statistical significance set at *P* < .05. We used the weighted χ2 test for categorical variables and the weighted linear regression model for continuous variables to calculate the difference among each group. The subgroup analysis was evaluated through stratified multivariable linear regression models. A weighted generalized additive model and smooth curve fitting were used to address the linear relationship between albumin and C-reaction protein.

## 3. Results

Our analysis included a total of 3579 participants whose age range from 60 to 80. The weighted characteristics and medical characteristics of the participants subclassified based on albumin quartiles (Q1:23–39 g/dL; Q2: 40–41 g/dL; Q3: 42–43g/dL; and Q4: 44–54 g/dL) were shown in Table 1. Among different groups of albumin (quartiles, Q1–Q4), age, alanine aminotransferase, blood urea nitrogen, creatinine, Gamma Glutamyl Transferase, CRP, body mass index, alkaline phosphatase, aspartate aminotransferase, cholesterol, creatine phosphokinase, globulin, lactate dehydrogenase, triglycerides, uric acid, race/ethnicity, martial status, smoking, high blood pressure, gender are all significantly different.

**Table 1:** Characteristics of the study population based on serum albumin quartiles.

Albumin g/dL	Q1	Q2	Q3	Q4	*P* value
N	746	768	870	1195	
Age (yr)	70.7 ± 7.3	70.2 ± 7.0	69.8 ± 6.9	69.2 ± 6.7	<.001
ALT (IU/L)	18.7 ± 12.6	19.9 ± 11.9	21.6 ± 12.8	23.6 ± 12.5	<.001
Blood urea nitrogen (mg/dL)	7.0 ± 3.4	6.4 ± 2.5	6.3 ± 2.3	6.0 ± 2.1	<.001
Creatinine (umol/L)	100.6 ± 71.9	87.2 ± 36.2	85.2 ± 36.7	82.8 ± 27.2	<.001
GGT (IU/L)	39.5 ± 72.2	29.3 ± 32.0	26.5 ± 32.1	28.6 ± 31.7	<.001
CRP (mg/L)	8.4 ± 14.8	4.5 ± 6.9	3.7 ± 5.4	2.6 ± 4.4	<.001
BMI (kg/m2)	31.1 ± 7.7	30.2 ± 6.9	29.7 ± 6.1	28.1 ± 5.0	<.001
ALP (IU/L)	91.2 ± 36.8	80.1 ± 24.9	77.1 ± 24.0	70.8 ± 20.2	<.001
AST (IU/L)	22.2 ± 14.1	22.2 ± 10.6	23.8 ± 11.2	25.5 ± 10.2	<.001
Cholesterol (mmol/L)	4.6 ± 1.1	4.9 ± 1.2	4.9 ± 1.1	5.0 ± 1.2	<.001
CPK (IU/L)	122.0 ± 116.1	130.7 ± 112.7	137.5 ± 141.8	154.1 ± 187.0	<.001
Globulin (g/L)	32.6 ± 5.5	30.4 ± 4.3	29.1 ± 4.1	27.9 ± 4.2	<.001
LDH (IU.L)	163.0 ± 45.3	153.8 ± 34.0	150.4 ± 34.5	144.8 ± 35.1	<.001
Uric acid (mg/dL)	5.9 ± 1.7	5.7 ± 1.6	5.6 ± 1.4	5.6 ± 1.3	<.001
Triglycerides (mmol/L)	1.5 ± 0.9	1.7 ± 1.0	1.8 ± 1.4	1.8 ± 1.2	<.001
Gender (%)					<.001
Male	335 (44.9%)	363 (47.3%)	428 (49.2%)	668 (55.9%)	
Female	411 (55.1%)	405 (52.7%)	442 (50.8%)	527 (44.1%)	
Race/ethnicity (%)					<.001
Non-**Hispanic** White	280 (37.5%)	312 (40.6%)	369 (42.4%)	497 (41.6%)	
Non-Hispanic Black	226 (30.3%)	177 (23.0%)	168 (19.3%)	183 (15.3%)	
Mexican American	98 (13.1%)	97 (12.6%)	116 (13.3%)	185 (15.5%)	
Other Hispanic	65 (8.7%)	89 (11.6%)	115 (13.2%)	151 (12.6%)	
Other **race**	77 (10.3%)	93 (12.1%)	102 (11.7%)	179 (15.0%)	
Martial status (%)					<.001
Living with partner	374 (50.1%)	422 (54.9%)	517 (59.4%)	732 (61.3%)	
Living without partner	371 (49.7%)	345 (44.9%)	351 (40.3%)	462 (38.7%)	
Don’t know	1 (0.1%)	1 (0.1%)	2 (0.2%)	1 (0.1%)	
Smoking (%)					.004
Yes	129 (17.3%)	84 (10.9%)	115 (13.2%)	148 (12.4%)	
No	271 (36.3%)	282 (36.7%)	296 (34.0%)	452 (37.8%)	
Don’t know	346 (46.4%)	402 (52.3%)	459 (52.8%)	595 (49.8%)	
High blood pressure (%)					.032
Yes	486 (65.1%)	445 (57.9%)	551 (63.3%)	704 (58.9%)	
No	259 (34.7%)	320 (41.7%)	317 (36.4%)	489 (40.9%)	
Don’t know	1 (0.1%)	3 (0.4%)	2 (0.2%)	2 (0.2%)	

Mean + SD, Sfor continuous variables: the *P* value was calculated by the weighted linear regression model. (%) for categorical variables: the *P* value was calculated by the weighted chi-square test.

ALT = alanine aminotransferase, ALP = alkaline phosphatase, AST = aspartate aminotransferase, BMI = body mass index, CRP = C-reactive protein, CPK = creatine phosphokinase, GGT = gamma glutamyl transferase, LDH = lactate dehydrogenase.

**Figure 1.**
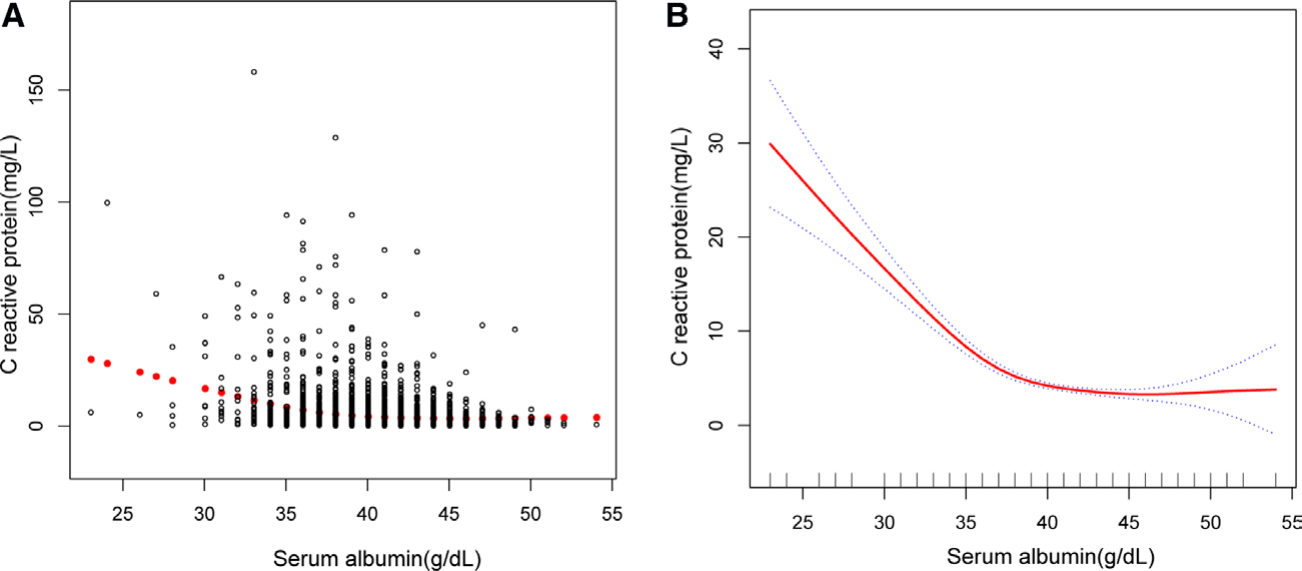
The association between albumin and C-reactive protein. (A) Each black point represents a sample. (B) Solid red line represents the smooth curve fit between variables. Blue dotted line represent the 95% of confidence interval from the fit. Age, gender, race/ethnicity, ALT, blood urea nitrogen, creatinine, GGT, BMI, ALP, AST, cholesterol, CPK, globulin, LDH, triglycerides, uric acid, martial status, smoking, high blood pressure were adjusted. ALT = alanine aminotransferase, GGT = gamma glutamyl transferase, BMI = body mass index, ALP = alkaline phosphatase, AST = aspartate aminotransferase, CPK = creatine phosphokinase, LDH = lactate dehydrogenase.

Three weighted multivariate linear regression models were constructed: model 1, not adjusted; model 2, age, gender, race/ethnicity were adjusted; model 3, the covariates presented in Table 1 were adjusted. In model 1, albumin was negatively correlated to CRP (β = −0.59, 95% CI: −0.66, −0.53, *P* < .0001). After adjusting confounders, the negative association was still present in model 2 (β = −0.60, 95% CI: −0.67, −0.53, *P* < .0001) and model 3 (β = −0.37, 95% CI: −0.45, −0.28, *P* < .0001) (Table 2). After converting albumin from a continuous variable to a categorical variable (quartiles), albumin level was also negatively associated with serum CRP in all groups (*P* for trend < .001 for each). Participants in the highest albumin quartile had a 2.86 times lower CRP than those in the lowest albumin quartile (Table 2). Smooth curve fittings and generalized additive models were used to characterize relationship between albumin and CRP (Fig. 1). The association was a slash. In the subgroup analysis stratified by gender, race/ethnicity, smoking, high blood pressure, the negative correlation of albumin with CRP was remained (Table 3). we also found that the level of CRP further decreased in other race (OR: −0.72, 95% CI: −0.96, −0.47 *P* < .0001) and participants with smoking (OR: −0.61, 95% CI: −0.86, −0.36 *P* < .0001).

**Table 2:** The association between albumin(g/dL) and C-reactive protein(mg/L).

Exposure	Model 1 β (95% CI) *P* value	Model 2 β (95% CI) *P* value	Model 3 β (95% CI) *P* value
Albumin(g/dL)	−0.59 (−0.66, −0.53) < .0001	−0.60 (−0.67, −0.53) < .0001	−0.37 (−0.45, −0.28) < .0001
Albumin categories			
Q1 (23–39 g/dL)	Reference	Reference	Reference
Q2 (40–41 g/dL)	−3.65 (−4.39, −2.91) < .0001	−3.65 (−4.38, −2.91) < .0001	−2.41 (−3.15, −1.68) < .0001
Q3 (42–43 g/dL)	−4.22 (−4.93, −3.50) < .0001	−4.23 (−4.94, −3.51) < .0001	−2.53 (−3.27, −1.79) < .0001
Q4 (44–54 g/dL)	−5.22 (−5.88, −4.55) < .0001	−5.24 (−5.91, −4.57) < .0001	−2.86 (−3.61, −2.11) < .0001
*P* for trend	<.001	<.001	<.001

Model 1: no covariates were adjusted; Model 2: age, gender, and race/ethnicity were adjusted; Model 3: age, gender, race/ethnicity. ALT, blood urea nitrogen, creatinine, GGT, BMI, ALP, AST, cholesterol, CPK, globulin, LDH, triglycerides, uric acid, martial status, smoking, high blood pressure.

ALT = alanine aminotransferase, ALP = alkaline phosphatase, AST = aspartate aminotransferase, BMI = body mass index, CPK = creatine phosphokinase, GGT = gamma glutamyl transferase, LDH = lactate dehydrogenase.

**Table 3:** Association between albumin (g/dL) and C-reactive protein (mg/L), stratified by sex, race/ethnicity, smoking and high blood pressure.

	Model 1 β (95% CI) *P* value	Model 2 β (95% CI) *P* value	Model 3 β (95% CI) *P* value
Sex			
Male	−0.67 (−0.77, −0.56) < .0001	−0.67 (−0.78, −0.56) < .0001	−0.42 (−0.55, −0.29) < .0001
Female	−0.54 (−0.62, −0.45) < .0001	−0.54 (−0.62, −0.45) < .0001	−0.34 (−0.44, −0.24) < .0001
Race/ethnicity			
Non-Hispanic white	−0.54 (−0.64, −0.44) < .0001	−0.56 (−0.66, −0.46) < .0001	−0.35 (−0.47, −0.24) < .0001
Non-Hispanic black	−0.71 (−0.88, −0.54) < .0001	−0.75 (−0.92, −0.58) < .0001	−0.37 (−0.56, −0.19) < .0001
Mexican American	−0.73 (−1.00, −0.46) < .0001	−0.73 (−1.01, −0.45) < .0001	−0.40 (−0.74, −0.07) < .0187
Other Hispanic	−0.56 (−0.79, −0.33) < .0001	−0.55 (−0.79, −0.32) < .0001	−0.40 (−0.70, −0.11) < .0080
Other race	−0.78 (−0.97, −0.58) < .0001	−0.78 (−0.98, −0.58) < .0001	−0.72 (−0.96, −0.47) < .0001
Smoking			
Yes	−0.69 (−0.89, −0.49) < .0001	−0.72 (−0.92, −0.51) < .0001	−0.61 (−0.86, −0.36) < .0001
No	−0.58 (−0.70, −0.46) < .0001	−0.58 (−0.70, −0.46) < .0001	−0.30 (−0.43, −0.16) < .0001
High blood pressure			
Yes	−0.64 (−0.74, −0.54) < .0001	−0.65 (−0.74, −0.55) < .0001	−0.42 (−0.53, −0.30) < .0001
No	−0.51 (−0.60, −0.42) < .0001	−0.52 (−0.61, −0.43) < .0001	−0.30 (−0.41, −0.19) < .0001

Model 1: no covariates were adjusted; Model 2: age, gender, and race/ethnicity were adjusted; Model 3: age, gender, race/ethnicity. ALT, blood urea nitrogen, creatinine, GGT,BMI, ALP, AST, cholesterol, CPK, globulin, LDH, triglycerides, uric acid, martial status, smoking, high blood pressure. In each case, the model is not adjusted for the stratification variable itself.

ALT = alanine aminotransferase, ALP = alkaline phosphatase, AST = aspartate aminotransferase, BMI = body mass index, CPK = creatine phosphokinase, GGT = gamma glutamyl transferase, LDH = lactate dehydrogenase.

## 4. Discussion

The main purpose of our study was to investigate whether albumin was independently related to CRP. In this study, we used a large nationally representative sample of American elders. The multivariate logistic regression analyses indicated an elevated albumin correlated with a lower CRP. Additionally, this decrease further reduced in other race and participants with smoking.

In the past few decades, malnutrition characterized by hypoalbuminemia is a common problem in older peoples.^[8,21,22]^ Hypoalbuminemia is not only a sign of malnutrition but also is negative consequences on most organs and systems.^[23]^ Meanwhile, It is significantly negatively correlated with the development of complications, mortality, inflammation and the average length of stay in acute patients.^[24,25]^ Identifying the presence and severity of inflammation is essential for assessing malnutrition. Cytokines, produced during inflammation, often result in anorexia, in large part by impairing the ability to digest or absorb nutrients.^[26]^ Hospitalized patients with severe inflammatory responses (defined as CRP > 100 mg/L) did not demonstrate a strong, measurable response to nutrition support in a large randomized controlled trial.^[27]^ Among our representative United States population, a lower albumin was associated with a greater CRP in old adults. Considering this association, hypoalbuminemia could provide a potential predictive biomarker for inflammation. Therefore, studying the relationship between albumin and CRP can provide a screening tool for inflammation to guide therapeutic intervention and avoid excessive correction of patients with inflammation.

Currently, there are limited clinical studies on the relationship between albumin and CRP in the elderly, and some of these studies are controversial. Two cross-sectional studies reported that peoples with hypoalbuminemia have elevated serum CRP levels comparing with normal urinemic patients.^[28,29]^ Ridker et al^[30]^ found that reduced albumin was associated with CRP when increased to values >13 mg/dL. Evans et al^[31]^ also reported that albumin characterize inflammation rather than describe nutrition status or protein-energy malnutrition. Both critical illness and chronic illness are characterized by inflammation and, as such, hepatic reprioritization of protein synthesis occurs, resulting in lower serum concentrations of albumin. Moreover, this paper has been approved by the American Society for Parenteral and Enteral Nutrition Board of Directors. However, partial studies came to the opposite conclusion and demonstrated that the albumin increased under systemic inflammation.^[32,33]^ Therefore, we further studied potential relationships and risk factors between albumin and CRP using a large sample.

The biggest strength of our study is that this study includes representative samples of the multiracial population and better universality of the United States population. Moreover, our large sample size allows us to perform subgroup analyses and is the first study, to our knowledge, to assess the association between albumin and CRP in different multivariate logistic regression models.

There are also some limitations in our study. First, our study is a cross-sectional design which limits the inference of a causal correlation between albumin and CRP among older peoples. Consequently, Further prospective studies with large study samples are needed to clarify the correlation between albumin and C-reactive protein in the elderly. Second, We have not adjusted for other potential confounding factors which may still lead to bias. Third, the age range of participants was 60 to 80, therefore, our conclusion cannot be generalized to the elderly over 80.

## 5. Conclusion

Our findings revealed that albumin levels was negatively associated with CRP levels among in USA elderly. Besides, CRP level decreased faster with increasing albumin level in other race and participants with smoking. Therefore, albumin should be correctly recognized as an inflammatory marker. Studying the relationship between albumin and CRP can provide a screening tool for inflammation to guide therapeutic intervention and avoid excessive correction of patients with inflammation.

## Author contributions

**Conceptualization:** Yiqian Jiang, Tiefeng Qiu.

**Data curation:** Yiqian Jiang, Jianhua Cao, Tiefeng Qiu.

**Formal analysis:** Tiefeng Qiu

**Investigation:** Qinghua Wu.

**Methodology:** Qinghua Wu.

**Resources:** Yiqian Jiang.

**Software:** Zhenli Yang.

**Supervision:** Zhenli Yang.
